# Accuracy of apical limit control during retreatment provided by hybrid electronic foraminal locators: A micro-CT study 

**DOI:** 10.4317/jced.62405

**Published:** 2025-01-01

**Authors:** Luciana Maria Arcanjo Frota, Raimundo Sales de Oliveira-Neto, Ana Grasiela Limoeiro, Murilo Priori Alcalde, Rodrigo Ricci Vivan, Marco Antônio Húngaro Duarte, Ricardo Affonso Bernardes, Bruno Carvalho Vasconcelos

**Affiliations:** 1 Post-graduate Program in Dentistry, Faculty of Pharmacy, Dentistry and Nursing, Federal University of Ceará, Fortaleza, CE, Brazil; 2 Department of Dentistry, Endodontics and Dental Materials, Bauru Dental School, University of São Paulo, Bauru, SP, Brazil; 3 Division of Endodontics, University of Maryland School of Dentistry Advanced Oral Science & Therapeutics, Baltimore, MD, USA

## Abstract

**Background:**

This study investigated the accuracy and reliability of apical limit control in endodontic retreatment using hybrid endodontic motors.

**Material and Methods:**

Thirty-six mesial canals of mandibular molars were accessed, and their apical foramina (AF) were standardized to 200 µm. Chemical-mechanical preparation was performed with WaveOne Gold instruments (#20/.07), followed by obturation with gutta-percha and AH Plus cement. After initial preparation, the teeth were divided into three groups (n = 12): Root ZX II (RZX), VDW Gold (VDW) and Tri Auto ZX2 (TRZX), all in rotary kinematics and with AF as the limit (0.0). Exposure was performed in the crown-down direction with ProTaper retreatment instruments using 2.5% NaOCl as an irrigation solution. Before using the instruments, the apexes of the teeth were immersed in alginate. With the auto-stop function activated, the hybrid instruments were calibrated to stop rotating when the desired apical limit was reached. The last instrument was fixed in the canal and a micro-computed tomography scan was performed to determine the distance between the instrument tip and the AF. Data were analyzed using the Kruskal-Wallis and Dunn tests (*P*<0.05).

**Results:**

No significant differences were found between the RZX (0.10 mm), Gold (0.13 mm) and TRZX (0.27 mm) devices; accuracy ranged from 91.70% (RZX and VDW) to 63.63% (TRZX). TRZX had the highest number of readings besides AF.

**Conclusions:**

It was concluded that all devices studied were efficient in maintaining the apical limit of instrumentation during endodontic retreatment when taken to the AF.

** Key words:**Endodontics, Electronic odontometry, Endodontic retreatment.

## Introduction

Accurate determination of the true canal length (TCL) and control of the apical limit during retreatment is crucial (1). This includes removal of the previous filling, cleaning, additional shaping and new filling of the canal system. The retreatment is the procedure of first choice in cases of failure of primary endodontic treatment (2-4). Errors can lead to inadequate cleaning or damage to the periapical tissue, hindering apical healing (5).

Studies support the use of electronic foraminal locators (EFLs) for TCL determination (6–10). Irrigation solutions such as sodium hypochlorite (NaOCl), ethylenediaminetetraacetic acid (EDTA), chlorhexidine and saline do not affect the EFL measurements (11,12). However, the effectiveness of EFLs can be influenced by gutta-percha, cements, and other materials (13,14). After removal of the filling material or chemical-mechanical preparation, the EFLs achieved accepTable values(15).

Hybrid devices that integrate EFLs with electric motors enhance procedure efficiency by monitoring root canal length in real time (6).

The Root ZX II (J. Morita, Tokyo, Japan) and VDW Gold (VDW GmbH, Munich, Germany) devices already known in the literature are examples of hybrid devices that use frequency-dependent impedance mechanisms at two frequencies. Both have already been evaluated for their accuracy in dynamic electronic odontometry during treatment or in certain treatment phases (6,8-10,16,17) but have rarely been tested in retreatment procedures.

The manufacturer of the Root ZX II, who already had a compact hybrid device, the Tri Auto ZX (J. Morita), has developed a new motor, the Tri Auto ZX2. This has similar technology to the Root ZX II, which is considered an accurate and reliable device (8-10). However, this time the inclusion of new functions and apical movements was included as a differential, such as Optimum Torque Reverse (OTR) and Optimum Glide Path (OGP), which would simplify the creation of a working path to the apex and determine the odontometry at the end, in addition to the rotary and reciprocal functions (9).

The lack of studies on the control of the apical limit of instrumentation promised by hybrid devices increases the interest in this topic. There are no reports in the literature evaluating the accuracy of these devices in controlling the apical limit during the removal of obturation material in endodontic retreatments.

The aim of this study was to evaluate the accuracy of control of the apical limit and the occurrence of readings beyond this limit by the Root ZX II, VDW Gold and Tri Auto ZX2 hybrid devices using rotary kinematics when taken to AF in endodontic retreatments. The null hypothesis tested was that the hybrid devices showed no significant differences in terms of precision and control of the apical limit of instrumentation during retreatment and no readings beyond the AF.

## Material and Methods

-Calculation and selection of samples

This study was approved by the local research ethics committee (#5,237,508) prior to its initiation. Sample size was determined using G*Power for Mac version 3.1 (Heinrich Heine; Fachhochschule Düsseldorf, Düsseldorf, Germany) using the Wilcoxon-Mann-Whitney test; the results of Vasconcelos *et al*. (6) were considered in this estimation. Thirty-six canals (n = 12) were then selected from mesial roots of Vertucci type IV human mandibular molars without marked dilatations (< 20°), with complete root formation, lengths between 18 and 20 mm, extracted for prosthetic, orthodontic and/or periodontal reasons, with open apical foramina and a diameter of less than 200 μm. A visual inspection and digital radiographs were taken to exclude teeth with previous endodontic treatment, fractures or calcifications; excluded teeth were replaced (9).

-Initial preparation of the specimens

All teeth were treated by a single surgeon with clinical experience. Endodontic access was performed with diamond tips No. 1014 and No. 3081 (KG Sorensen, Cotia, Brazil), which were driven at high speed with irrigation. Flat surfaces were created on the occlusal part of the teeth, which served as a safe point for positioning the penetration limiters of the endodontic instruments. The patency of the foramina was checked with 25 mm type-K #10 hand files (Dentsply/Sirona, Ballaigues, Switzerland), and the CRC of the teeth was determined using a clinical microscope (16x) until the tip of the file could be seen through the AF. Canals with an AF of more than 150 μm (confirmed by the passage of a Nitiflex #15 type-k file) were replaced. After foraminal standardization, which was performed with a Nitiflex #20 type-k file, specimens were numbered, and chemical-mechanical preparations were performed with a WaveOne Gold Small (#20/.07) instrument (Dentsply-Maillefer) driven by a VDW Gold electric motor in the WaveOne All program.

Instrumentation was performed in the order recommended by the manufacturer with slight forward and backward movements of up to 0.0 mm, using the previously established RCL as a parameter. The instrument was cleaned every three movements, and the canal was irrigated with 1.0 mL of 2.5% NaOCl (Biodinâmica, Ibipora, Brazil), which was introduced into the canal using an endodontic syringe and special needles (Navitip; Ultradent, South Jordan, USA).

The canals were dried with absorbent paper cones (Dia-pro W Small; Diadent, Burnaby, Canada) and obturation was performed using the single-cone technique, with the apical margin set at 1.0 mm below the AF; #25/.06 WaveOne Gold primary percha cones (Dentsply-Maillefer) wrapped with AH Plus cement (Dentsply-Maillefer) were used. After vertical condensation and cleaning of the access cavity, the teeth were sealed with a temporary cement (Coltosol; Vigodent, Bonsucesso, Brazil) and the samples were x-rayed and stored in an oven (Quimis, Diadema, Brazil) at 100 % humidity and 37 °C for 21 days (18).

-Classification of the test groups and analysis of the apical limit

The teeth were randomly divided into three groups according to the equipment used: Root ZX II (RZX), VDW Gold (GOLD) or Tri Auto ZX2 (TRZX); in this randomization, the lengths and curvatures were considered. The specimens were mounted in groups of 4 teeth on a special holder, with the root apices immersed in alginate (Jeltrate II; Dentsply, Petrópolis, Brazil). The operator, who had been previously calibrated, performed the exposure with all devices programmed for the auto-stop function at the 0.0 limit (apex) in the crown-apex direction using the ProTaper Universal Retreatment instruments (Dentsply-Maillefer).

The three files of the system were used at the same speed and torque standards (300 RPM and 2.0 N), with slight apical pressure movements, until the last D3 instrument (#20/.07) reached the point set by the device’s automatic shut-off mechanism. After each sequence of three picks, the instrument was removed, cleaned in gauze, and the canal irrigated again with 1.0 mL of 2.5% NaOCl. When the D3 instrument reached the apical limit of instrumentation determined by the hybrid device, its penetration was interrupted and it was separated from the contra-angle handpiece and fixed to the coronal part of the specimen using a cyanoacrylate-based adhesive (Super Bonder; Loctite do Brazil, São Paulo, Brazil) (6).

-Microtomography scans and analyzes

The teeth were scanned in a microtomograph (SkyScan 1174v2; Bruker-microCT, Kontich, Belgium) with 70kV, 800 mA, 360º rotation with 0.1 steps around the vertical axis, a voxel size of 9.88 mm, an isotropic resolution of 19.6 µm, a rotation of 0.7°; an average pixel size of 2000 x 1332 and a thickness of 1 mm aluminum filter, with an instrument inserted into the root canal space. The digital images were reconstructed using NRecon v1.6.4.8 software (Bruker-microCT) and provided transverse and axial slices in BMP format.

For each measurement, the measurement error was calculated as the difference in millimeters between the tip of the instrument and a tangent crossing the edges of the greater foramen. Positive and negative values were recorded when the tip was detected above and below the tangent, respectively. Accuracy was measured in mm using FIJI/ImageJ software (Fiji v.1.51n; Fiji, Madison, WI).

-Statistical analysis

The data were subjected to the Kolmogorov-Smirnov test to check the normality of the data, which confirmed the absence of a normal distribution. The results were then analyzed using the Kruskal-Wallis test, followed by Dunn’s individual comparison tests, assuming a significance level of 5%. In addition, the frequency with which the devices offered readings beyond the AF was analyzed using the chi-square test with a significance level of 5%. The GraphPad Prism software was used for the statistical comparisons.

## Results

**Figure 1:**
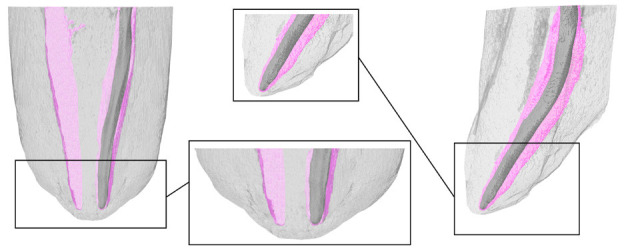
Representative micro-CT image of the electronic measurement analysis.

A representative picture of the results can be seen in Figure 1.  Table 1 shows the medians and ranges of the mean errors found for the groups, considering the absolute values. No significant differences were found between the hybrid devices tested (*P*>0.05).  Table 2 shows the distributions of the positions of the instrument tip in relation to the determinations at 0.0 mm and the frequency of accurate readings below and outside the AF. RZX and GOLD showed an accuracy of 91.70% when considering the percentages of accurate and accepTable specimens within a tolerance of 0.5 mm. In addition, the chi-square test revealed that the TRZX had a significantly higher number of readings beyond the AF compared to the RZX (*P*<0.05).

**Figure 2:**
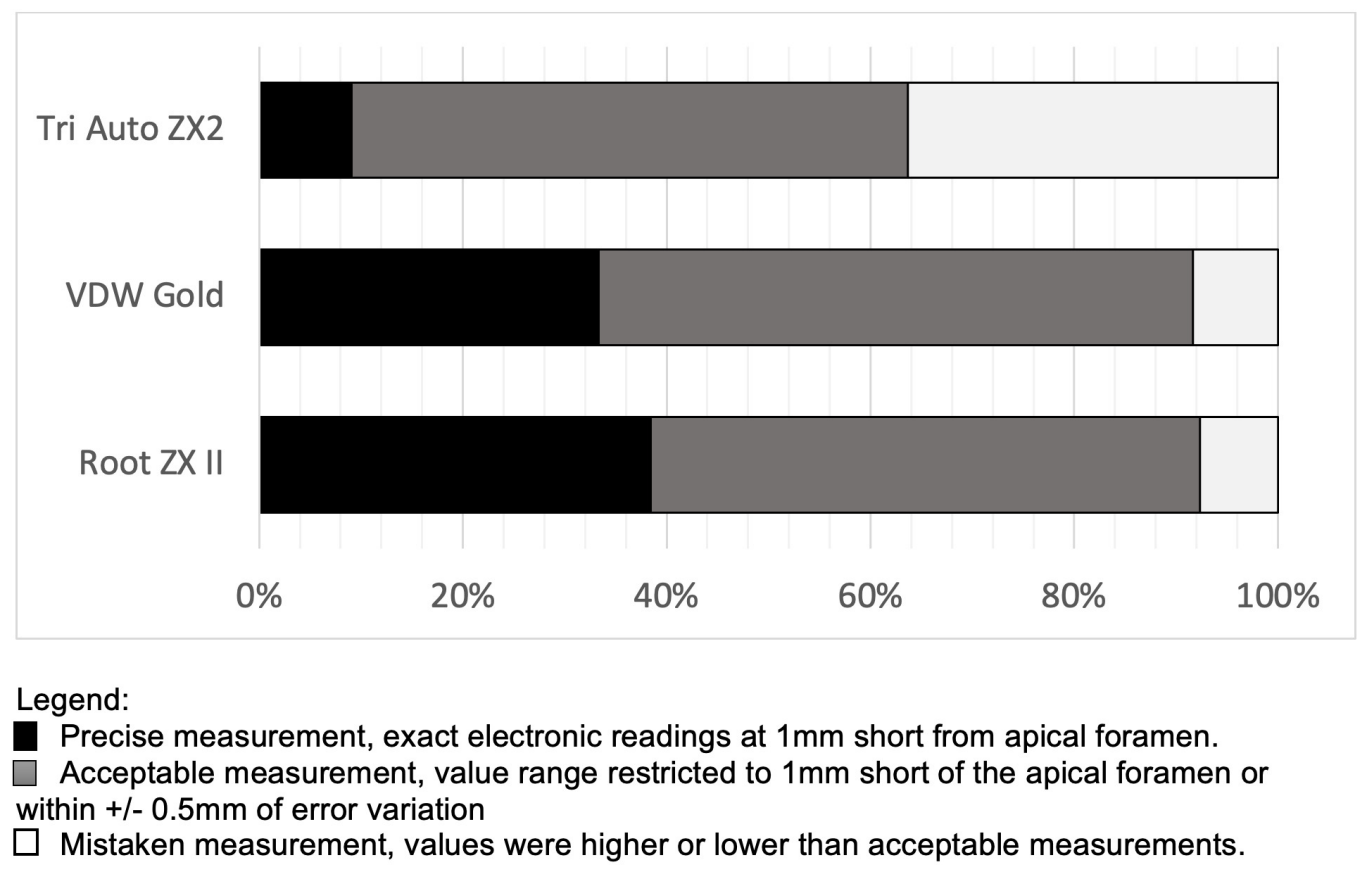
Percentage of precise, acceptable, and mistaken readings found in hybrid equipment.

The graphical representation of the percentages corresponding to accurate, acceptable and incorrect measurements of the hybrid devices can be seen in Figure 2.

## Discussion

In this study, the performance of the RZX, GOLD and TRZX hybrid devices in endodontic retreatment was evaluated. Considering the importance of correctly determining the working length in these cases, especially given the risk of not correctly cleaning the entire length of the canals or assuming the possibility of leakage of filling material or contaminated dentin. To date, there have been no studies that have evaluated the control of the apical limit in hybrid EFLs when determining WL in retreatments. Considering the results, the hypothesis of the study was not accepted as the TRZX had a greater number of readings than the AF.

The methods used here follow similar parameters to previous studies that used the alginate model, prior cervical widening and the use of instruments adjusted in the apical third (6,7,9,19,20).

The assessment technique used was micro-CT, which is considered an important tool for investigating the accuracy of EFLs compared to conventional methods such as visual inspection (21), scanning electron microscopy (22) and digital photographs with a clinical microscope (6). Micro-CT, although expensive, provides detailed information about SCRs and is currently the best technique for this type of study (23–25). In this study, the display of the 0.0 mm marker on the screen of the hybrid LEFs, referred to as the foramen major in the literature, was considered as a reference point, as it is easily recognizable on the micro-CT images and its position can be repeatedly reproduced (25).

The devices tested proved to be accurate, considering the average error values found to be between 0.10 mm (RZX) and 0.23 mm (TRZX). This result contrasts with some studies in which it was found that even conventional EFLs show interference in the presence of filling materials such as gutta-percha and cement, causing reading errors (21,26). It could be suggested that the accuracy of the electronic devices was favored by the protocols for the preparation of the canals and the use of the devices that corresponded to the clinical conditions under which the EFLs are more accurate. This allowed the accuracy of the devices to be very close to the values found in the literature for their use in the initial instrumentation of canals (7,9).

Regarding the results of the individual devices, a more direct comparison is hampered by the fact that there are no previous studies evaluating the accuracy of the automatic system of the electronic hybrid devices during the clearance procedure in retreatments. However, in general, the RZX is the device most frequently used as a reference in comparative studies and usually provides excellent results (6,9,19,24,25). This result was repeated in the present study, with the RZX showing the lowest error results (0.10 mm) and the highest accuracy (91.7%), but not significantly different from the others in terms of average error. Like the RZX, the GOLD also reproduced accuracy parameters similar to those observed with treatments, i.e. without gutta-percha and cement (7).

TRZX, like the others, had a very low average error (0.27 mm), but 36.36% of the readings were outside the AF and outside the tolerance of 0.5 mm. This high rate can be explained by the time that elapses between the length detection by the hybrid EFL and the automatic stopping of the movement by the auto-stop function at limit 0.0 (27). Measurements beyond the foramen may also have a direct relationship with the speed of the devices and the pressure exerted by the operator. When analyzing its predecessor, only one study evaluated the TRZX in endodontic retreatment and showed an accuracy of more than 80% of teeth within +/-0.5 mm after the removal of root fillings from upper and mandibular canines (28), however, the device was not used during instrumentation.

Undoubtedly, one of the advantages of hybrid devices is the continuous monitoring of WL, as its variations during cervical preparation and RCS instrumentation have already been consolidated in the literature (29,30). Therefore, it is necessary to maintain control over the apical limit throughout the treatment, not only in the odontometry phase, due to the possibility of reducing the expansion of the canals after shaping the canals, especially in curved canals such as those used in this study. (29,30) If WL reduction is not considered, the risk of overextension of the preparation and filling increases, leading to failures in endodontic treatment that may jeopardize its success. Although the results of this study must be extrapolated with caution to clinical practice, the observations draw attention to the possibility of using hybrid preparation devices in the removal of fillings, with RZX and GOLD being safer, while in the case of the TRZX there is a greater risk of exceeding the apical foramen during retreatment.

## Conclusions

The Root ZX II, VDW Gold and Tri Auto ZX2 hybrid devices were found to be accurate in controlling the apical limit of instrumentation during endodontic retreatment when taken up to the apical foramen. In addition, the accuracy of the Root ZX II, VDW Gold was over 90%. However, the Tri Auto ZX2 not only provided slightly lower accuracy, but also had a greater number of readings beyond the apical foramen.

## Figures and Tables

**Table 1:** Distance (mm) from device measurements to 0.0.

Devices	Median	Margin	
		Min	Max
Root ZX II	0,10ª	0,00	0,55
Tri Auto ZX2	0,27ª	0,00	0,88
VDW Gold	0,13ª	0,00	0,78

Max, maximum; Min, minimum. Different superscript lowercase letters indicate statistically significant differences between different devices in the same position according to Dunn’s test (*P*< 0.05). *Median calculated in terms of absolute values of determinations.

**Table 2:** Position of the file tip in relation to the apical foramen, measurements taken at 0.0.

Distance from apical foramen (mm)	Root ZX II		VDW Gold		Tri Auto ZX2	
	n	%	n	%	n	%
< -0,51*	0	0	0	0	0	0
-0,5 to -0,01	4	33,3	2	16,7	0	0
0,0	5	41,7	4	33,3	1	9,09
0,01 to 0,5	2	16,7	5	41,7	6	54,54
> 0,51	1	8,3	1	8,3	4	36,36

* The negative value indicates the position of the file below the apical foramen.

## Data Availability

The datasets used and/or analyzed during the current study are available from the corresponding author.
